# Biodegradable Pansy® occluder for patent foramen ovale closure: a multicenter, single-arm, prospective study

**DOI:** 10.3389/fcvm.2025.1464712

**Published:** 2025-04-04

**Authors:** Lu He, Hang Xie, Yongsheng Gao, Gangcheng Zhang, Qunshan Shen, Minghua Wang, Qiguang Wang, Yujiu Wang, Yajuan Du, Xianyang Zhu, Yushun Zhang

**Affiliations:** ^1^ Department of Structural Heart Disease, Xi'an Jiaotong University Medical College First Affiliated Hospital, Xi'an, Shaanxi, China; ^2^ Department of Cardiac Surgery, The First Bethune Hospital Jilin University, Changchun, Jilin, China; ^3^ Department of Cardiac Surgery, Wuhan Asia Heart Hospital, Wuhan, Hubei, China; ^4^ Department of Cardiac Surgery, Shandong Province Qianfushan Hospital, Jinan, Shandong, China; ^5^ Department of Congenitial Heart Disease, General Hospital of Northern Theater Command, Shenyang, Liaoning, China; ^6^ Department of Cardiac Surgery, Binzhou Medicial University Hospitial, Yantai, Shandong, China

**Keywords:** patent foramen ovale, biodegradable, occluder, interventional, therapy

## Abstract

**Background:**

The next-generation closure device for interventional treatment of patent foramen ovale (PFO) is regarded as biodegradable, yet the corresponding biomaterial technique is still challenging. Herein, we report the clinical application of a novel biodegradable PFO occluder [made of the biodegradable material polydioxanone (PDO)] that is finally coming into clinical use.

**Objectives:**

This study aimed to assess the safety and efficacy of the biodegradable Pansy® occluder (Mallow Medical, Shanghai, China) for PFO closure in patients exhibiting PFO with a substantial right-to-left shunt (RLS).

**Methods:**

Six centers in China participated in this prospective, multicenter study of PFO closure from June 2019 to September 2020. Serious adverse events occurring in the perioperative period and during follow-up were systematically collected. Contrast transthoracic echocardiography (cTTE), transthoracic echocardiography (TTE) and transesophageal echocardiography (TEE) were performed during the preoperative and follow-up periods.

**Results:**

A total of 137 patients with a mean age of 38.1 ± 12.4 years who underwent catheter-based PFO closure with the biodegradable Pansy® occluder were included. The procedural success rate was 99.3%. Except for 2 cases (1.4%) of micropericardial effusion, there were no other complications such as cardiac tamponade, major bleeding, stroke oroccluder embolization. During the 12-month follow-up, serious adverse events occurred in 3 patients (2.2%), all of which were device-related thrombus (DRT). Four patients (2.9%) still had moderate to substantial residual RLS. The complete occlusion rate was 97.1% at 12 months after closure.

**Conclusions:**

PFO closure with the biodegradable Pansy® occluder can be performed effectively with acceptably low complication rates, low occurrence of adverse events, high procedural success rates and high complete occlusion rates at follow-up.

**Clinical Trial Registration:**

http://www.chictr.org.cn/index.aspx; identifier (ChiCTR1900024036).

## Introduction

Over the past 7 years, multiple randomized controlled trials (RCTs) have established robust evidence for preventing cryptogenic stroke (CS) by percutaneous closure of patent foramen ovale (PFO) (1–4). Therefore, the Amplatzer PFO occluder, as a double-disk, NiTi alloy occluder developed in the 1990s, is indicated for percutaneous closure of a PFO to reduce the risk of recurrent ischemic stroke in patients, predominantly between the ages of 18 and 60 years, who have had CS due to a presumed paradoxical embolism, as determined by a neurologist and cardiologist following an evaluation to exclude known causes of ischemic stroke. Nevertheless, similar to the drawbacks of NiTi alloy occluders in the transcatheter treatment of congenital heart disease (CHD), there are still some limitations in terms of PFO NiTi alloy occluders, including device-related thrombus (DRT), arrhythmia, late erosion of the occluder, nickel allergy and other factors (5–8). In addition, the PFO NiTi alloy occluders may interfere with future septal procedures, such as atrial septal puncture. Based on the concept that cells can sense and adapt to their surroundings, modification of the surface of a biomaterial becomes an important way to improve the performance of an implant. Nevertheless, these modifications toward NiTi alloy occluders still cannot change the fact that metal occluders exist *in vivo* permanently.

In view of the problems mentioned above, a biodegradable PFO occluder is urgently needed considering that PFO occluders contribute to a large proportion ofcurrent clinical applications.We screened biodegradable polymer materials and finally selected biodegradable polydioxanone (PDO) wire as the double-disk skeleton of the occluder and polyglycolic acid (PGA) as the suture thread. The bluff body still retains the traditional polyethylene terephthalate (PET) polyester film. Different from the structure of the Amplatzer PFO occluder, the metal rivet on the left disk is removed to reduce the probability of thrombosis and speed up endothelialization. Accordingly, this prospective, multicenter clinical study reported the initial assessment of the safety and efficacy of the biodegradable Pansy® occluder in patients exhibiting PFO with a substantial right-to-left shunt (RLS).

## Methods

### Study design

The present trial was a prospective, nonrandomized, multicenter study. Enrollment was started in June 2019 and finished in September 2020. The trial was registered in the Chinese Clinical Trial Registry (http://www.chictr.org.cn/index.aspx; ChiCTR1900024036) and approved by theethics committee of the First Affiliated Hospital of Xi'an Jiaotong University (XJTU1AF2019LSY-44). The study was reviewed by the China National Medical Products Administration and approved by each site's institutional review board. Informed consent was obtained from all participants. An independent data and safety monitoring boardmet periodically to assess safety and trial integrity. The authors vouch for the completeness and accuracy of the data and analyses and for thefidelity of the trial to the protocol. Clinical evaluations, including physical examination, chest radiography, electrocardiography (ECG), transthoracic echocardiography (TTE),contrast transthoracic echocardiography (cTTE)/contrast-enhanced transcranial Doppler (cTCD) and transesophageal echocardiography (TEE), were performed before the procedure.

### Study population

Between June 2019 and September 2020, 138 patients who underwent PFO closure were prospectively enrolled at 6 hospitals in China (The First Affiliated Hospital of Xi'an Jiaotong University, Wuhan Asia Heart Hospital, General Hospital of Northern Theater Command, The First Bethune Hospital Jilin University, Shandong Province Qianfushan Hospital, Binzhou Medicial University Hospitial).

Inclusion criteria: (1) PFO with substantial RLS; (2) Age greater than 16 years; (3) Voluntary participation in the clinical trial and signing of the informed consent form. Exclusion criteria: (1) Known causes of ischemic stroke; (2) Contraindications to antithrombotic therapy; (3) Systemic or local infection, sepsis, intracardiac thrombosis, complete obstruction of the inferior vena cava; (4) Pregnancy; (5) Pulmonary hypertension or PFO with a special channel; (6) Massive cerebral infarction within 4 weeks; (7) Inability to sign informed consent. All patients at each center who satisfied the inclusion criteria and did not meet the exclusion criteria were included in this study.

### Echocardiography protocols and definitions

A GE-ViVid-E9 color Doppler ultrasound system (General Electric Corporation, Norfolk, VA) equipped with a 2∼4 MHz transducer was used to perform TTE, and a 4∼7 MHz transducer was used to conduct TEE. All patients had to undergo TEE examination to confirm the diagnosis of PFO and to evaluate its anatomical characteristics. For TTE and TEE, all the right atrium and interatrial septal characteristics other than PFO were recorded, including atrial septal aneurysm (ASA), membrane mobility >6.5 mm (9), tunnel length, prominent Eustachian valve (EV), Chiari's network, and presence or absence of an atrial septal defect (ASD), as well as its size if present.

The severity of the shunt is a subjective assessment performed by cTTE/cTCD. Agitated saline was used as the contrast agent and consisted of 8 ml of saline solution, 1 ml of air, and 1 ml of blood collected from the patient. Contrast agent was injected into the left cubital vein as a bolus. When using cTTE, the apical four-chamber view was generally selected. The presence of RLS was confirmed when microbubbles (MBs) were seen in the left atrium within the first 3 cardiaccycles after MB appearance in the right atrium at rest or after the Valsalva maneuver (VM). According to Li Yue, et al. (10), theseverity of RLS was semiquantified into a four-level scale, and >30 MBs were defined as substantial RLS. According to current standards (11), cTCD with a TCD monitoring device (DWL MultidopX, ScanMed Medical, Gloucestershire, UK) can also be used to identify the degree of RLS. Both middle cerebral arteries were simultaneously monitored through the temporal window using 2 MHz probes. The contrast agent was used as mentioned above. The presence of RLS was confirmed when high-intensity transient signals (HITS) appeared within 25 s after contrast agent injection at rest or after VM. The severity of RLS was semiquantified into a four-level scale, and we defined substantial RLS as shower or curtain HITS.

### Characteristics of the biodegradable pansy® occluder

**Figure 1:**
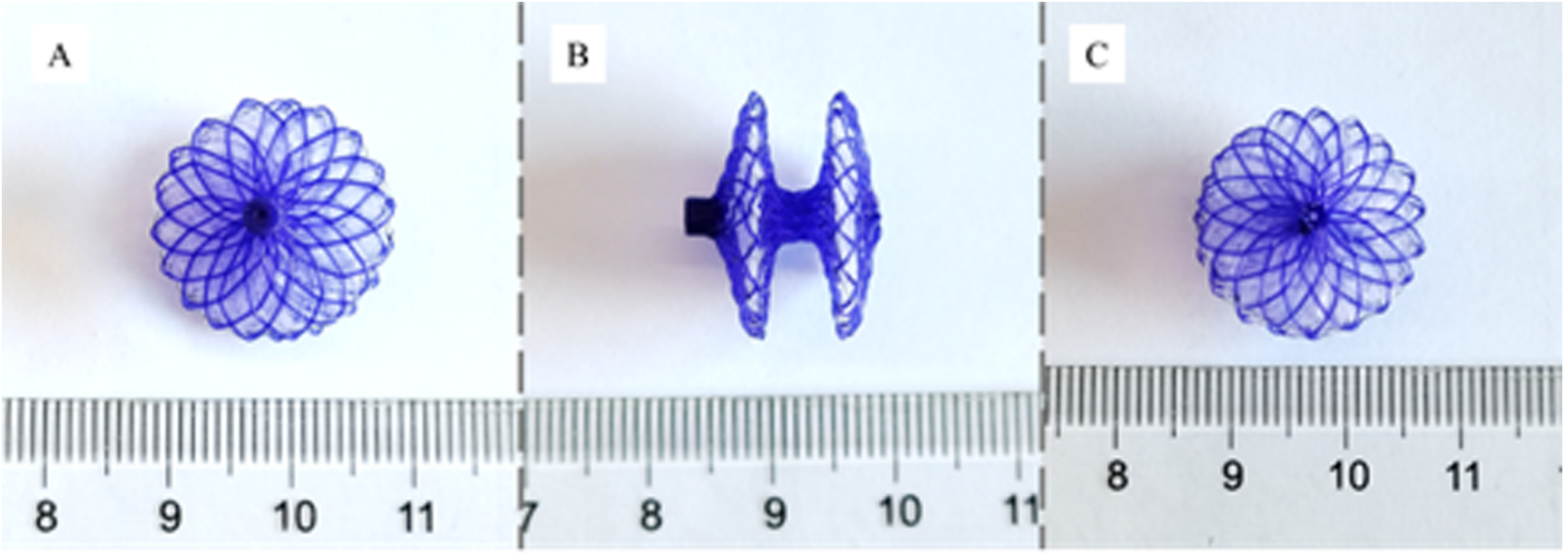
Biodegradable pansy® occluder. **(A)** Right atrium side view; **(B)** profile view; **(C)** left atrium side view.

**Figure 2:**
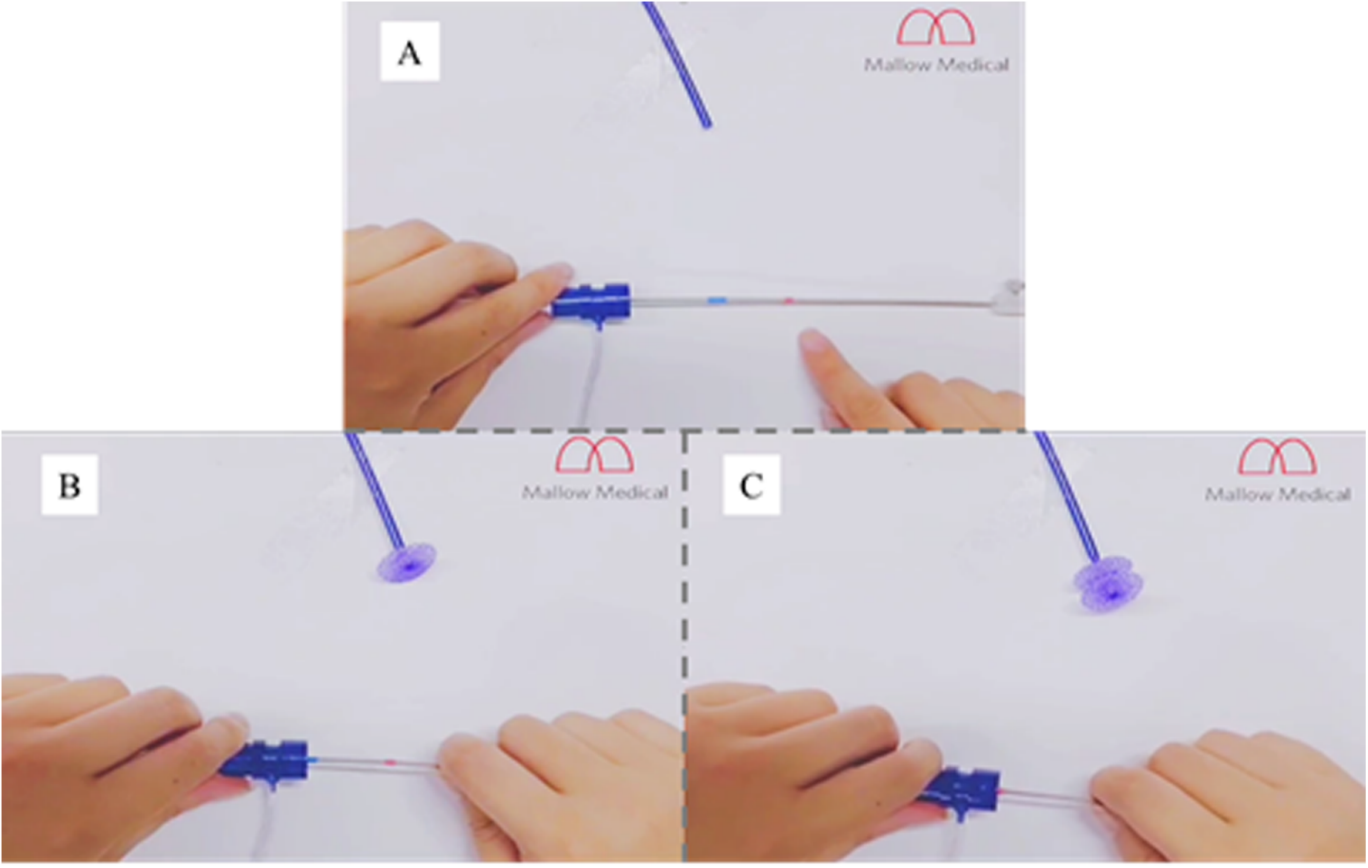
Specially made conveying cable. **(A)** The blue and red marking points correspond to the position of the left disc and right disc of the occluder after release. **(B)** The blue marking point correspond to the position of the left disc of the occluder after release. **(C)** The red marking point correspond to the position of the right disc of the occluder after release.

The device is a self-expanding PDO wire tube that consists of 2 discs, each with a sewn polyester patch and a central connecting waist (4 mm). Different from the structure of the Amplatzer PFO occluder, the metal rivet on the left disk is removed to reduce the probability of thrombosis and speed up endothelialization (Figure 1). There are 7 sizes available: 18/18 mm, 24/18 mm, 24/24 mm, 30/24 mm, 30/30 mm, 34/24 mm and 34/34 mm. The device size corresponds to the diameter of the right and left disc of the occluder. The occluder is delivered through a dedicated sheath using a deliverycable that is attached to the proximal end of the occluder via an end screw. The 18/18 mm, 24/18 mm and 24/24 mm devices can be delivered through a 10-Fr delivery sheath, whereas the 30/24 mm, 30/30 mm, 34/24 mm and 34/34 mm devices require a 12-Fr delivery sheath. There are two marking points distal to the conveying cable, which correspond to the position of the left disc and right disc of the occluder after release (Figure 2).

### Percutaneous closure of PFO

**Figure 3:**
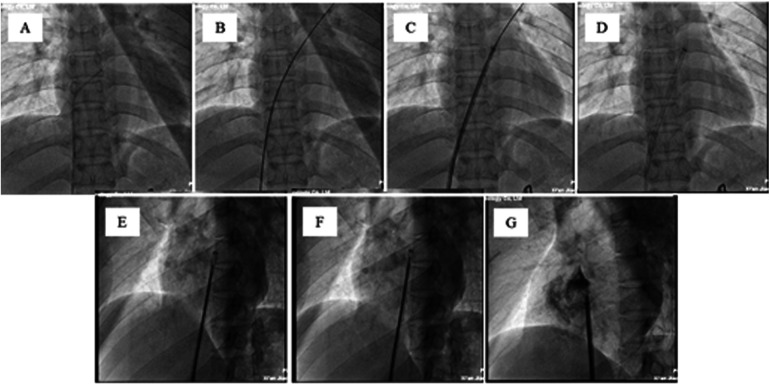
Transcatheter closure of the PFO with a biodegradable pansy® occluder. **(A)** A multipurpose (MP) diagnosticcatheter (6-Fr) is advanced over the wire to the base of the right atrium (RA). The catheter is directed towardthe interatrial septum (IAS), a maneuver that can be assisted by aligning the orientation of the MP catheter with the direction of theimaging element of the fossa ovale angiography. **(B)** A standard 0.035-inch J-tipped wire is advanced through the MP catheter across the PFO and into the left upper pulmonary vein (LUPV). **(C)** A 0.035-inch Amplatzer extrastiff guidewire (260 cm) is advanced over the MP catheter to the LUPV. Then, the delivery sheath is advanced over the 0.035-inch Amplatzer extra stiff guidewire to the left atrium. **(D)** The expansion sheath and the 0.035-inch Amplatzer extra stiff guidewireare withdrawn. **(E)** Under TTE guidance, the left disc of the occluder is opened and located in the left atrium. **(F)** Under TTE guidance, the right disc of the occluder is opened, and the occluder is located on the IAS. G. After the diluted contrast medium is delivered through the sheath, the occluder can be seen in good position, and the left and right discs are clearly separated.

**Figure 4:**
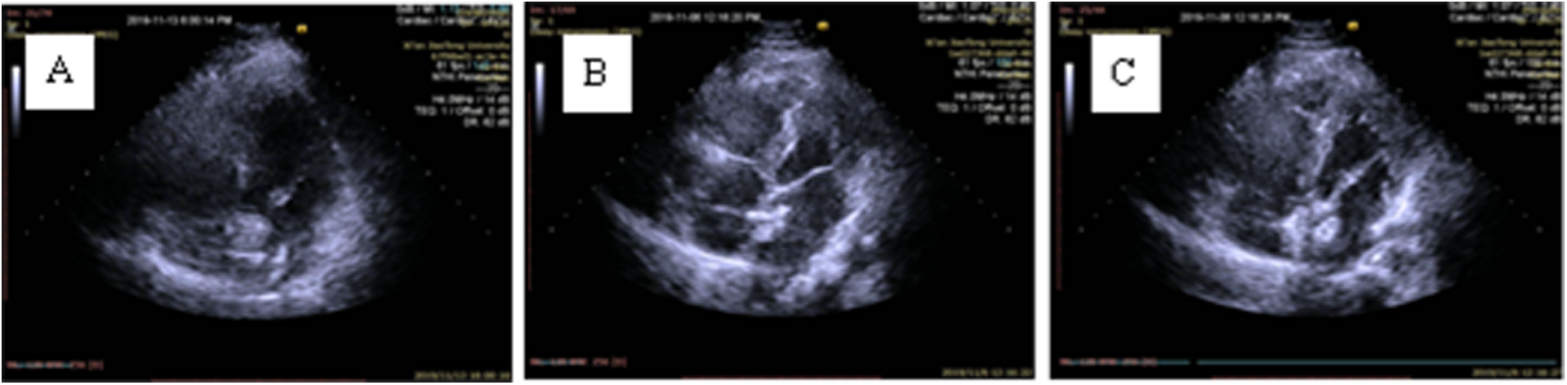
The release process of the occluder under TTE guidance. **(A)** The left disc is deployed in the left atrium; **(B)** The left disc gradually fits the interatrial septum (IAS); **(C)** The right disc is released.

The procedure was performed with fluoroscopic and transthoracic echocardiography (TTE) guidance under general anesthesia via a femoral vein approach. Heparin was administered according to the patient's body weight (80∼100 IU/kg) to achieve an ACT >250 s. Transcatheter closure of PFO with a biodegradable Pansy® occluder is shown in Figure 3 and Figure 4. All patients were treatedwith low-molecular-weight heparin at 10 U/(kg·h) for 48 h, aspirin 100 mg/day for 6 months, and clopidogrel 50∼75 mg/day for 3 months following device implantation.

### Follow-up

Clinical follow-up was performed at 24 h, 1month, 3 months, 6 months and 12 months after the procedure. At each visit, detailed 2D-TTE was performed to assess the device position and residual shunt, and ECG was recorded to evaluate cardiac arrhythmia. TEE was performed at 6 months to evaluate the size and position of the occluder. cTTE was followed up at 6 months after the procedure to observe residual RLS (rRLS). If there was no rRLS, cTTE was not required in future follow-up exams. If the RLS remained, cTTE and TEE examinations were performed at the 12-month follow-up. For patients with symptoms of palpitation and chest pain, Holter monitoring and ECG were needed at every follow-up exam.

### Statistical analysis

Descriptive data for continuous variables are presented as the mean ± SD. Categorical variables are presented as relative frequencies. Given the descriptive nature of this study and the small sample size, no between-group comparison was performed. All statistical analyses were performed with commercially available software (PASW Statistics v20.0.0; SPSS, Inc., Chicago, IL).

## Results

### Study population and preprocedure data

There were 138 patients enrolled in the study in 6 cardiology centers in China. A total of 137 patients who underwent successful catheter-based PFO closure with the biodegradable Pansy® occluder were included. One patient diagnosed as PFO with a small ASD, the device closured the small ASD and retained the PFO, was not included in the statistical analysis. Their baseline characteristics are shown in Table 1. The mean patient age was 38.1 ± 12.4 years, and 37.2% were men. The degree of RLS was evaluated by cTTE and/or cTCD. cTTE was performed in 125 (91.2%) cases, while cTCD was performed in 90 (65.7%) cases. A total of 78 (56.9%) patients completed both cTTE and cTCD examinations. The specific use of cTTE or cTCD was at the discretion of each center. According to the current consensus and guidelines of PFO in various countries (12–19), patients who undergo PFO closure need to have medium to substantial RLS. Therefore, this study only included patients with substantial RLS at rest and/or after VM by cTTE/cTCD. The details are shown in Table 2.

**Table 1:** Baseline characteristics.

Variable	PFO closure cohort (*n* = 137)
Demographics	
Mean age, years	38.1 ± 12.4
Male, *n* (%)	51 (37.2%)
PFO-related clinical syndromes	
CS, *n* (%)	17 (12.4%)
TIA, *n* (%)	3 (2.2%)
Migraine, *n* (%)	94 (68.6%)
OSAHS, *n* (%)	1 (0.7%)
Others, *n* (%)	22 (16.1%)
Preprocedure echocardiographic findings	
PFO size at rest (RA side)	1.7 ± 0.6
PFO size at rest (LA side)	1.1 ± 0.6
PFO size after VM (RA side)	4.2 ± 1.7
PFO size after VM (LA side)	1.9 ± 0.7
PFO tunnel length	8.2 ± 3.0
Combining small ASD, *n* (%)	1 (0.7%)
ASA or membrane mobility > 6.5 mm, *n* (%)	7 (5.1%)
Chiari's network, *n* (%)	2 (1.5%)
Prominent EV, *n* (%)	1 (0.7%)
Combining bicuspid aortic valve, *n* (%)	2 (1.5%)

PFO, patent foramen ovale; CS, cryptogenic stroke; TIA, transient ischemic attack; OSAHS, obstructive sleep apnea-hypopnea syndrome; RA, right atrium; LA, left atrium; VM, valsalva maneuver; ASD, atrial septal defect; ASA, atrial septal aneurysm; EV, eustachian valve.

**Table 2:** cTTE/cTCD assessment of RLS perioperatively.

Test status	RLS	cTTE (*n* = 125)	cTCD (*n* = 90)
At rest, *n* (%)	0	68 (54.4%)	40 (44.4%)
	Ⅰ	32 (25.6%)	21 (23.3%)
	Ⅱ	14 (11.2%)	15 (16.7%)
	Ⅲ	11 (8.8%)	14 (15.6%)
After VM, *n* (%)	Ⅰ	1 (0.8%)	2 (2.2%)
	Ⅱ	9 (7.2%)	8 (8.9%)
	Ⅲ	115 (92.0%)	80 (88.9%)

cTTE, contrast transthoracic echocardiography; cTCD, contrast-enhanced transcranial Doppler; RLS, right-to-left shunt; VM, valsalva maneuver.

### Procedural results

Successful device implantation was achieved in 137 (99.3%) patients. The size distribution of the successfully implanted devices is shown in Table 3. No complications occurred during the procedure. As of September 2021, all 137 patients (100%) completed the 12-month follow-up. At the 6-month follow-up, 136 patients (99.3%) underwent TEE examination on time; 1 patient (0.7%) underwent TEE examination at 8 months; at the 12-month follow-up, 137 (100%) patients completed cTCD/cTTE examination on time.

**Table 3:** Procedure characteristics.

Variable	PFO closure cohort (*n* = 137)
Occluder size, mm	
24/18	2 (1.5%)
24/24	128 (93.4%)
30/30	4 (2.9%)
34/34	3 (2.2%)
Procedural success, *n* (%)	137 (100%)
Procedural complication, *n* (%)	0 (0%)

### Clinical and echocardiographic follow-up

Clinical follow-up at 24 h, 1month, 3 months, 6 months and 12 months was available for all patients (100%). During the 12-month follow-up, a total of 3 patients (2.2%) suffered serious adverse events, all of which were DRT. The complete occlusion rate was 97.1% (133/137) at 12 months after the procedure.

**Figure 5:**
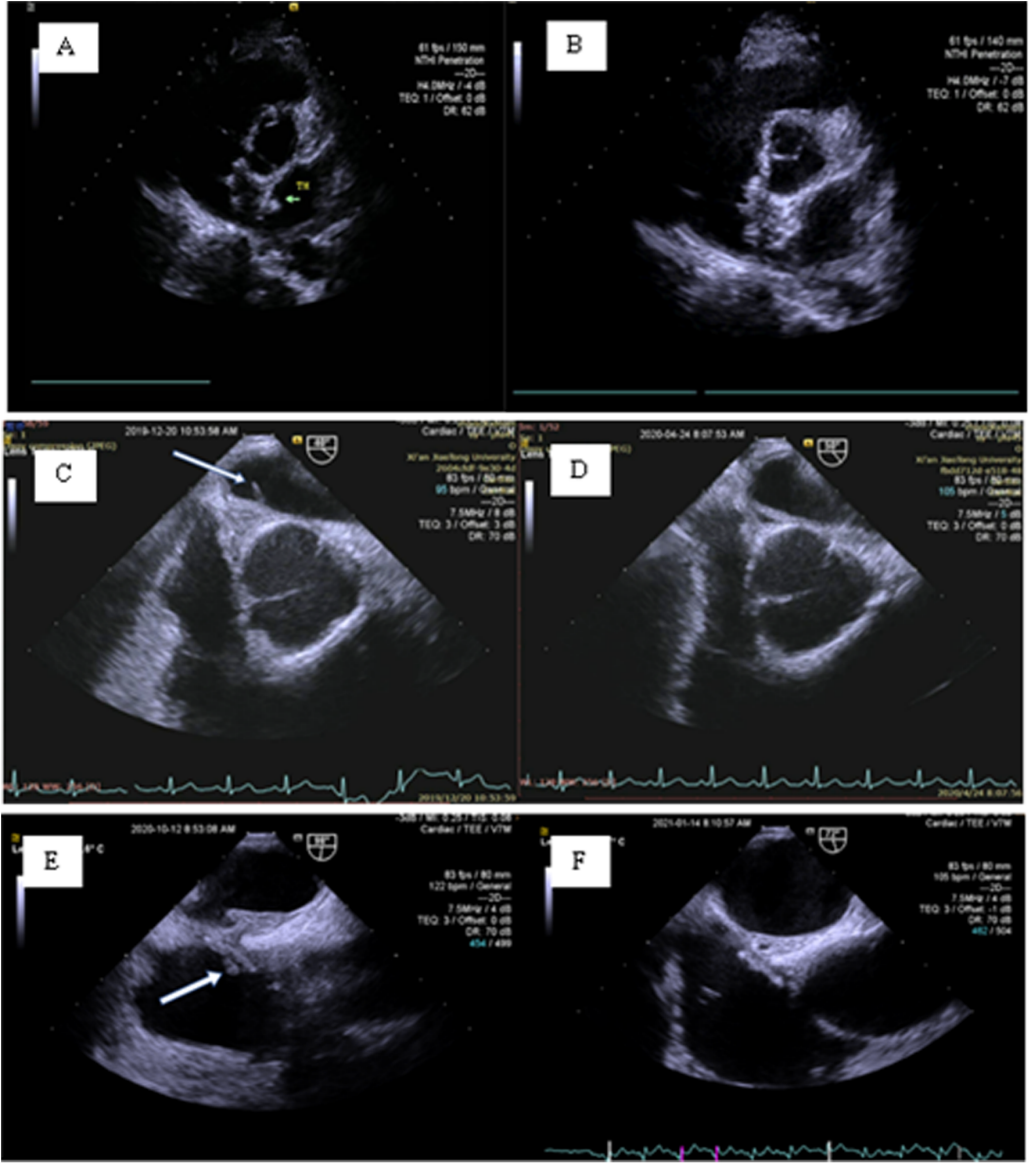
Postprocedure DRT. **(A,B)** A 42-year-old female: At the 3-month follow-up, TTE revealed a thrombus on the left disc of the occluder (green arrow). **(C,D)** A 45-year-old female: At the 6-month follow-up, TEE revealed an abnormal cord-like echo (7.4  ×  1.7 mm) on the left disc of the occluder (white arrow). The follow-up medical history revealed that the patient was hit by a stone on her foot approximately 20 days after the procedure, and a red bruise appeared under the skin. The patient discontinued aspirin and clopidogrel by herself and concealed the history of discontinuation during the 3-month follow-up. **(E,F)** A 38-year-old female: At the 6-month follow-up, TEE revealed an abnormal strip of shadow on the right disc of the occluder (white arrow).

One case of DRT was located on the left disk of the occluder at the 3-month follow-up by TTE. The other two cases of DRT were found atthe 6-month follow-up by TEE. For all of the above patients, rivaroxaban 20 mg/day was immediately given orally, and TEE re-examination after 1 month of treatment showed that the DRT disappeared. The specific DRT situation is shown in Figure 5.

**Figure 6:**
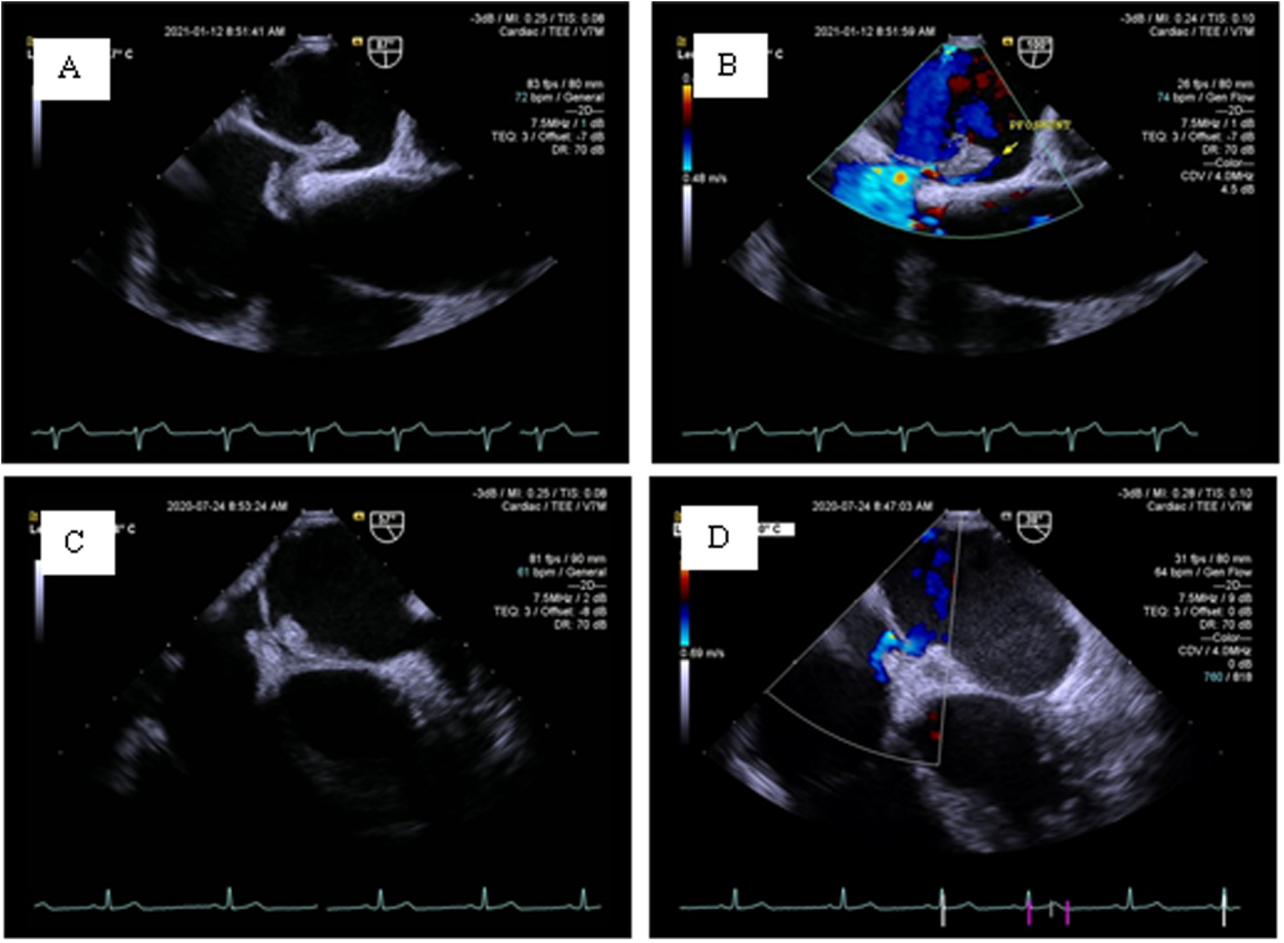
TEE examination of medium to substantial rRLS. **(A,B)** A large PFO (4.5 mm) with a long tunnel (13.5 mm) was implanted with a 30/30 mm PFO occluder. The left disc was degraded to 8 mm and did not completely cover the left side of the PFO. A tunnel-like channel was formed in the occluder. **(C,D)** A PFO with a small ASD; the device achieved closure of the PFO and retained the small ASD. On 2D-TEE, the small ASD was not clearly displayed. Color Doppler flow imaging showed a transseptal shunt through the small ASD.

At 48 h, 1month, 3 months, 6 months and 12 months post-procedure follow-up, TTE examination showed that all137 patients (100%) had no left-to-right shunt (LRS). At the 6-month follow-up, cTTE/cTCD examination showed that 4 patients (2.9%) had a small amount of RLS after VM, 27patients (19.7%) still had medium to substantial rRLS after VM, and the remaining 106 patients (77.4%) had no rRLS. Further analysis found 8 cases (5.8%) of rRLS at the pulmonary arteriovenous level. Although the remaining 19 cases (13.9%) of rRLS were medium to substantial, the amount of RLS was significantly reduced compared with the preoperative state. Among the above mentioned patients with medium to substantial rRLS, only 4 patients (2.9%) had medium to substantial rRLS after VM when the cTTE/cTCD was re-examined at the 12-month follow-up. TEE was performed in4 patients with medium to substantial rRLS at the 12-month follow-up. Detailed information is shown in Figure 6.

In addition, 2 cases (1.5%) of asymptomatic micropericardial effusion were observed, one of them occurring within 48 h after the procedureand the other case occurring intermittently between 1 month and 6 months after the procedure. Two patients (1.5%) in this study developed postoperative new-onset premature atrial beats, which were both incidental and transient, with no obvious discomfort and no need for antiarrhythmic treatment.

## Discussion

This study is the first multicenter evaluation of percutaneous PFO closure with the degradable Pansy® closure system. The device was successfully implanted in 99.3% of the patients, and the complete occlusion rate was 97.1% with no major complications in the perioperative period and a low rate of serious adverse events (2.2%) at the 12-month follow-up.

The BioSTAR occluder was the first occluder to propose the concept of “degradable”. The BioSTAR septal repair implant (NMT Medical, Boston, Mass) was a novel, bioabsorbable device specifically designed for the closure of ASDs and PFOs. BioSTAR used an acellular porcine intestinal collagen layer (ICL) matrix (Organogenesis, Canton, Mass) mounted on an MP35N STARFlex (NMT Medical) “double-umbrella” framework. Ultimately, the BioSTAR occluder was withdrawn from the market due to the high probability of thrombosis and the fracture of the skeleton and similar structure of the STARFlex occluder (20, 21).

Since biodegradable occluders are regarded as next-generation closure devices, various attempts have been made to develop biodegradable devices for CHD closure. To our knowledge, the Pansy® PFO occluder is the first biodegradable PFO occluder to be used in the human body.

Due to the poor memory performance of degradable ASD occluder materials and the need for additional locking devices, the clinical operation is relatively complicated. However, the PDO material has better shape memory function, and the shape of the biodegradable Pansy® occluder is similar to that of the Amplatzer double-disc PFO occluder. Therefore, the operation is relatively simple and completely similar to conventional transcatheter PFO closure. Therefore, the transcatheter interventional closure procedure are easy, and the learning curve is short.

Since the occluder is not visible under x-ray, we add special marking pointsdistal to the conveying cable, which correspond to the position of the left disc and waist of the occluder after release. Then, we can complete the operation under the guidance of x-ray and TTE. To maximize the patient benefit, this study included patients with a substantial RLS at rest and/or after VM by cTTE/cTCD examination, which greatly improved the safety and reduced the complication of the procedure.

DRT is a rare but severe complication of PFO closure. Abaci et al. (22) conducted a meta-analysis of more than 28,000 patients and found that the incidence of DRT on PFO occluders was approximately 2.5% to 13.3%. Moreover, the incidence of DRT was related to whether TEE was performed during follow-up, and small thrombi may not have been accurately observed by TTE. Our study protocol required all patients to undergo TEE examination at the 6-month follow-up. During the follow-up period, a total of 3 cases of DRT were found, the incidence of which was similar to that of a previous NiTi alloy occluder. Fortunately, none of the patients had stroke. One case was related to the patient's self-discontinuation of the drug and the lack of regular antiplatelet therapy after the procedure; the other two cases occurred during conventional dual antiplatelet therapy. One month after anticoagulant therapy was administered, all the above DRT disappeared. Therefore, regular and effective antithrombotic therapy after PFO closure can minimize DRT events. Thrombosis caused by the implantation of degradable materials may be related to slight differences in the rate of disintegration of the degradable polymer matrix surface. The occluder has a certain chance of developing DRT before it is adequately covered by the neo-endothelium. Early degradable stents also have DRT events. In this study, the incidence of DRT was relatively high. On the one hand, it was determined by the characteristics of the PDO material itself during the degradation process. On the other hand, due to the early discontinuation of antiplatelet drugs in individual patients, DRT was formed before endothelialization. Therefore, for the postoperative medication of the degradable Pansy® occluder, a sufficient amount and a full course of antiplatelet therapy should be emphasized. Whether it is necessary to prolong the antiplatelet time or directly use anticoagulation therapy requires long-term follow-up results with a large sample size. In the course of clinical follow-up, once DRT is found or highly suspected, close follow-up is required, and sufficient anticoagulant drug treatment should be used immediately to prevent the adverse events of thrombus shedding that leads to embolism of other organs.

rRLS is the most common complication after PFO closure, which can occur in or around the occluder. A small amount of rRLS is more common after PFO closure, and medium to substantial rRLS is rare. Caputietc (23) reported that the incidence of rRLS at 6 months and 12 months after PFO closure was 19.5% and 18.2%, respectively. However, the incidence of substantial rRLS after 12 months was less than 3%, which is consistent with our study. The current study found that the type of occluder is closely related to rRLS after PFO closure, and the Amplatzer PFO occluder has the lowest incidence of rRLS (24). At present, it is believed that the reasons for the occurrence of rRLS are as follows. First, the early rRLS is related to the type and characteristics of the occluder. The principle of PFO closure is to pull the primary septum to the secondary septum, and the probability of rRLS before the early endothelialization of the occluder is high. Second, medium to substantial rRLS are mostly related to the missed diagnosis of other defects in the preoperative examination. In this study, two cases of medium to substantial rRLS both involved a PFO with a small ASD; the device achieved closure of the PFO and retained the small ASD. Third, the structural characteristics of PFO are also related to rRLS. PFO with ASA and tunnel length > 20.8 mm are independent predictors of rRLS after PFO closure at 12 months (25). In this study, 3 cases of long-tunnel PFO or PFO combined with membrane mobility >6.5 mm had substantial rRLS after PFO, all of which were poorly fit between the disc and the atrial septum, and PFO remained after degradation, resulting in substantial rRLS. Since the hardness of the degradable material is lower than that of Nitinol, after implantation of the degradable occluder, the primary septum and the secondary septum cannot obtain enough tension to fit each other, which may not be suitable for PFO with tunnels that are too long. Closure device, especially for long-tunnel PFO, a degradable occluder may not be the right choice. Therefore, a detailed preoperative measurement of the PFO anatomical characteristics can play a key role in reducing the occurrence of rRLS.

Pericardial effusion is not uncommon in cardiac interventional therapy. In this study, 2 cases of asymptomatic micropericardial effusion were observed, one of which occurred within 48 h after the procedure and was considered to be related to intervention therapy. Another case occurred intermittently between 1 month and 6 months after the procedure. TTE/TEE examination did not reveal any abnormality of the occluder. The effusion was considered to be unrelated to the occluder, and the reason was unclear. In general, these 2 cases of micropericardial effusion did not cause clinical symptoms and resolved on their own without special treatment.

In addition, a total of 2 patients in this study developed postoperative new-onset premature atrial beats, which were both incidental and transient, with no obvious discomfort and no need for antiarrhythmic treatment. Similarly, transient atrial arrhythmias have been reported after Amplatzer PFO occluder implantation and generally do not require special treatment. We believe that the reason for the low incidence of atrial arrhythmia after degradable occluder implantation is related to the study design, arrhythmias due to nickel allergy caused by previous metal occlusions cannot be ignored (26–28). Atrial arrhythmia after PFO closure can only be detected by implanting a loop recorder or long-term Holter monitoring. However, in our study, ECG was only performed at the postoperative follow-up period, so the incidence of atrial arrhythmia after closure was unknown. However, the low incidence of atrial arrhythmias at postoperative follow-up may be an advantage of biodegradable materials, and more rigorous studies with large sample sizes dedicated to atrial arrhythmias are needed to prove this.

### Limitation

This study also had certain limitations. Although it was a prospective, multicenter clinical trial, since there is currently no biodegradable PFO occluder available for clinical application in China, this study did not set up a parallel control of biodegradable occluders, nor did it set up a randomized control of NiTi alloy occluders, such as the Amplatzer PFO occluder. In addition, the main purpose of this study was to assess the safety and efficacy of the biodegradable Pansy® occluder for PFO closure in PFO patients with substantial RLS. The evaluation of the efficacy on headache and CS was not included in this study. Therefore, cohort studies conducting comparisons with drug treatment have not been set up. Finally, the follow-up endpoint of this study was only set to 12 months, and longer observational studies will be required in the future to evaluate the safety and efficacy of this device.

## Conclusion

In this multicenter study, we demonstrated the safety and efficacy of the biodegradable Pansy® occluder for PFO closure in patients exhibiting PFO with substantial RLS. Multicenter experience has shown that the use of degradable occluders for transcatheter PFO closure has a high success rate and a low incidence of adverse events. The most common postoperative complications were DRT and rRLS. It is suggested that the degradable PFO occluder can be safely used in the clinic. Careful preoperative assessment, standardized transcatheter interventional closure procedure, and selection of appropriate occluders can reduce adverse events.

## Data Availability

The original contributions presented in the study are included in the article/Supplementary Material, further inquiries can be directed to the corresponding author.
